# *Ex vivo* quantification of intracellular pH in *Drosophila* Malpighian tubule reveals basolateral HCO_3_^−^/oxalate exchange through a novel oxalate transporter “Neat”

**DOI:** 10.3389/fphys.2025.1468451

**Published:** 2025-04-28

**Authors:** Adam J. Rossano, Lili Zhang, Jacob B. Anderson, Heather L. Holmes, Asim K. Mandal, James W. Decker, David B. Mount, Michael F. Romero

**Affiliations:** ^1^ Physiology and Biomedical Engineering, Mayo Clinic College of Medicine and Science, Rochester, MN, United States; ^2^ Renal Division, Brigham and Womens Hospital and Harvard Medical School, Boston, MA, United States; ^3^ Biomedical Engineering, Boston University, Boston, MA, United States; ^4^ Renal Division, West Roxbury VA Medical Center, West Roxbury, MA, United States; ^5^ Nephrology and Hypertension, Mayo Clinic College of Medicine and Science, Rochester, MN, United States

**Keywords:** oxalate transport, *Drosophila* Slc26, bicarbonate transport, genetically encoded pH-sensor, renal epithelia, intracellular pH, Malpighian tubule

## Abstract

**Introduction:**

Nephrolithiasis is a painful and costly healthcare complication. The most common kidney stones are composed of calcium oxalate and thus renal handling of oxalate is an important facet of understanding the pathogenesis of nephrolithiasis. Recently, the *Drosophila melanogaster* Malpighian tubule (MT) has emerged as a robust model of trans-epithelial ion transport and nephrolithiasis as MTs readily form luminal calcium-oxalate crystals in the presence of oxalate. *Drosophila* Prestin (dPrestin, Slc26a6) transports oxalate across the apical surface of the MT into the lumen but a full model of the trans-epithelial movement of oxalate (Ox^2−^) in the *Drosophila* MT has been lacking as the basolateral oxalate transporter has remained uncharacterized.

**Methods:**

The objective of this work was to identify and characterize the *Drosophila* basolateral Ox^2−^ transporter through *ex vivo* real-time quantification of intracellular pH (pH_i_) and *Xenopus* oocyte transport assays.

**Results:**

A putative basolateral oxalate transporter CG5002 (“Neat”) was identified through sequence homology and displayed robust Cl^−^-independent Ox^2−^ transport and electroneutral Ox^2−^ transport in *Xenopus* oocytes. pH_i_ in extracted fly MTs was monitored by using the GAL4/UAS system to selectively express pHerry, a pseudo-ratiometric genetically-encoded pH indicator (GEpHI) in the cytosol of the principal cells of the MT. Basolateral perfusion of MTs in CO_2_/HCO_3_^−^-buffered solution produced a large acidification followed by rapid recovery in the transitional segment of the anterior MT. Recovery was interrupted by basolateral application of 1 mM Ox^2−^ or 1 mM SO_4_^2^. Tissue specific knock-down of Neat with interference RNA (RNAi) reduced the rate of acid-loading in the transitional segment of the MT with regard to Ox^2−^ and SO_4_^2−^. Knockdown of Neat in the MT also significantly reduced luminal calcium oxalate crystal formation in a fly *ex vivo* model of calcium oxalate nephrolithiasis.

**Discussion:**

These data indicate Neat is a significant *Drosophila* basolateral MT oxalate transporter and the basolateral movement of oxalate is functionally coupled to movement of acid equivalents, potentially as Ox^2−^/HCO_3_^−^ exchange, Ox^2−^/OH^−^ exchange, or Ox^2−^:H^+^ co-transport.

## 1 Introduction

Nephrolithiasis is a worldwide disease (∼10%) with an increasing prevalence and significant morbidity, which is affected by both environmental and genetic factors (Wang and Wang, 2024). It is characterized by the formation of various crystals within the kidneys or urinary tract. The most common form of nephrolithiasis results from calcium oxalate (CaOx) deposition. The mechanism of CaOx formation in nephrolithiasis involves multiple factors, among which the imbalance between inhibitors and promoters of Ca^2+^ and oxalate (Ox^2-^) transepithelial transport and crystallization is of potential importance. Understanding the mechanisms of those anion movements is crucial for developing strategies to prevent and treat CaOx-related kidney stones. Therefore, an assessable model of transepithelial Ox^2-^ movement is desirable.

The adult *Drosophila melanogaster* (fruit fly) Malpighian renal tubule (MT) is a highly active excretory organ lined with a single epithelial layer of metabolically active principal cells. It has been identified as a site of rapid calcium excretion (Dube et al., 2000; MacPherson et al., 2005; Southall et al., 2006; Terhzaz et al., 1999) and Ox^2−^ transport (Chen et al., 2011; Hirata et al., 2012a; Hirata et al., 2010), similar to the functions of the human kidney. *Drosophila* reliably develop CaOx crystals upon dietary exposure to high (10 mM) Na_2_Ox supplementation and the growth of microliths in MT can be viewed in real time. Cellular assays of ion transport physiology can be combined with genetic binary expression systems to target fluorescent reporter and RNA interference (RNAi) constructs to individual cells, thus providing a robust system for studies of solute transport at the molecular, cellular, and organ scales. Therefore, our lab and others developed a *Drosophila* model of CaOx nephrolithiasis which can be used for more precisely investigating ion and solute movements (Chen et al., 2011; Hirata et al., 2012a; Hirata et al., 2010). Using this model, we have identified *Drosophila* prestin (dPrestin), a key apical transporter which mediates Ox^2−^ and SO_4_^2−^ export into MT lumen. Formation of CaOx crystals quantitatively decreased in dPrestin-knockdown animals (Hirata et al., 2012a; Hirata et al., 2012b). These data, as well as studies of oxalate transport via dPrestin in a *Xenopus* oocyte expression system, indicate dPrestin is functionally the *Diptera* ortholog of SLC26A6 and mouse Slc26a6 (Landry et al., 2016). These proteins play important roles in the transporting multiple anions, e.g., Cl^−^, HCO_3_^−^, Ox^2−^, SO_4_^2−^, and formate^−^ (Landry et al., 2016; Ohana et al., 2013; Shimshilashvili et al., 2020; Yang et al., 2021).

Prior results indicate that dPrestin functions as an apical Ox^2−^ transporter in fly MTs, but how oxalate is moved from the hemolymph into the MT to complete the model of transepithelial Ox^2−^ transport has not been determined. In mammals, a sulfate anion transporter (Sat1, Slc26a1) was cloned by functional expression from rat liver as a Na^+^ independent sulfate transporter (Bissig et al., 1994). Subsequent work demonstrated oxalate and HCO_3_^−^ transport via Slc26a1 (Karniski et al., 1998; Xie et al., 2002). Oocyte expression experiments illustrated pH-dependent sulfate uptake (Xie et al., 2002). Based on their expression work, Krick and associates hypothesized that in the proximal tubule under physiological conditions, Slc26a1 (Sat1) exchanges intracellular sulfate for extracellular HCO_3_^−^ which in turn is recycled by Slc4a4 (NBCe1) to contribute to kidney oxalate secretion in hyperoxalemic patients (Krick et al., 2009). SO_4_^2−^ uptake was competitively inhibited by Ox^2−^ (Ki of 63.5 µM), a value close to the oxalate concentration detected in the serum of patients with hyperoxaluria or end-stage renal disease, whereas oxalate uptake showed saturation kinetics with a Km of 53.5 µM, a value close to that obtained for the Ki for competitive inhibition of SO_4_^2−^ uptake by Ox^2−^ (63.5 µM), indicating an interaction at the same site of SLC26A1 (Krick et al., 200). Genetic knock-out experiments in mice revealed that sat1 (Slc26a1)-loss is associated with hyposulfatemia, hypersulfaturia, calcium oxalate urolithiasis, and nephrocalcinosis (Dawson et al., 2010; Markovich, 2011). Human SAT1 (SLC26A1) mutations are associated with calcium oxalate nephrolithiasis (Gee et al., 2016). Thus, in mammals, both apical Slc26a6 and basolateral Slc26a1 affect the CaOx kidney stone formation. This makes it important to understand how these apical and basolateral transporters work together to regulate transepithelial oxalate transport and subsequent CaOx stone formation.

Similarly, for transepithelial Ox^2−^ transport in the *Drosophila* Malpighian tubule (MT), there should be another Ox^2−^ transporter. This yet unknown transporter should ideally be at the basolateral membrane to move Ox^2−^ from the hemolymph into the MT, so that dPrestin might then move Ox^2−^ from the MT cell to the MT lumen to then be excreted. The aim of the current study was to identify another *Drosophila* oxalate transporter, elucidate its function, and determine if it impacts calcium oxalate (CaOx) crystallization in this model.

**FIGURE 1:**
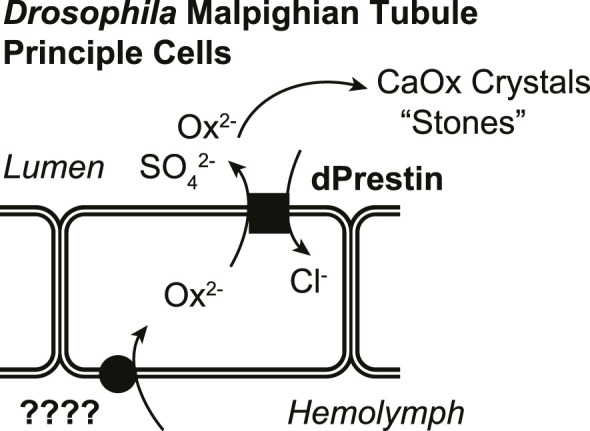
Rationale for model of oxalate transport in *Drosophila* Malpighian tubule principal cells.

In a functional simplified *in vivo* model (*Drosophila*), knowing both apical and basilateral transporters will facilitate understanding differences and similarities of CaOx crystallization-stone formation (Figure 1). Here, we have characterized a *Drosophila* Ox^2−^ transporter (CG5002; “Neat”; named such as knockdown produces MTs “without rocks”) as a candidate for the other oxalate transporter. These studies use *Xenopus* oocyte expression to test Ox^2−^ transport by *Drosophila* Slc26 member, and then the GAL4/UAS system (Brand and Perrimon, 1993) to selectively express pHerry (Rossano et al., 2013), a genetically-encoded pH indicator (GEpHI), with and without RNA interference (RNAi)-mediated knockdown of Neat while also following MT crystal formation. Together these data support the hypothesis that Neat is likely a *Drosophila* oxalate uptake system working in concert with dPrestin, is bicarbonate coupled, and is a functional equivalent of mammalian basolateral Ox^2−^ transporter Slc26a1.

## 2 Methods

### 2.1 Ethical approval - animal health and welfare

*Xenopus laevis* were housed and cared for in accordance and approval of the Institutional Care and Use Committees of the Mayo Clinic College of Medicine and Brigham and Women’s Hospital.

### 2.2 RNA and oocyte care

Constructs containing coding regions of indicated transporters were subcloned into the pGEMHE *Xenopus* expression vector and capped cRNA synthesized. Oocytes were injected with 50 nL cRNA (0.2 μg/μL, 10 ng/oocyte) or water as previously (Hirata et al., 2010; Romero et al., 2000) for other transporters and incubated at 16°C in OR3 media. Oocytes were studied 3–10 days after injection. Amount of cRNA injected was constant through all experiments and transporter expression was assumed to be constant as well.

### 2.3 Oocyte oxalate uptake assay

All uptake experiments using *Xenopus laevis* oocytes were performed as described previously (Mandal et al., 2017; Mandal and Mount, 2019). Briefly, for [^14^C]-oxalate uptake experiments in oocytes, each oocyte was microinjected using a fine-tipped micropipette fitted in a microinjector (World Precision Instruments Inc. Sarasota, FL) with equal amount (∼25 ng/50 nL) of *in vitro* synthesized intact appropriate cRNA (checked by 1% formaldehyde-agarose gel electrophoresis in MOPS buffer, pH 8.0) and incubated in isotonic ND96 medium (96 mM NaCl, 2.0 mM KCl, 1.8 mM CaCl_2_, 1.0 mM MgCl_2_ and 5 mM HEPES, pH 7.4) containing 3 mM pyruvate and gentamycin (10 μg/mL at 16°C–18°C for approximately 48 h for the expression of respective protein from injected cRNA) in chloride-free medium (Cl^−^ was substituted by equimolar gluconate ion), pH 7.4 at 25°C. Oocytes were then washed four times with isotonic ND96 medium without pyruvate and gentamycin. After 1 h of incubation in the indicated chloride-free uptake medium (1 mL/well) containing [^14^C]-oxalate (20 μM; 51 μCi/μmol; Moravek Inc. Brea, CA) in 12-well plates at room temperature [25°C] in a horizontal shaker-incubator, oocytes (15–20 in each group) were washed three times with the ice-cold uptake medium to remove external radioisotope. Radioisotope content of each individual oocyte was measured by scintillation counter (LS 6500 multi-Purpose Scintillation Counter, Beckman Coulter, Brea, CA) after solubilization in 0.3 mL of 10% (v/v) SDS and addition of 2.5 mL of scintillation fluid. All uptake experiments included at least 15–20 oocytes in each experimental group; statistical significance was defined as p ≤ 0.05 by one-way ANOVA with Bonferonni’s multiple comparisons tests and results were reported as mean ± SD. All of the uptake experiments were performed more than three times for confirmation with appropriate controls; data shown for each figure are from a single representative experiment (units are pmol/oocyte/h).

### 2.4 Oocyte electrophysiology

Electrophysiology protocols were performed as we previously reported (Romero et al., 2000). All solutions were either ND96 (96 mM NaCl, 2 mM KCl, 1.8 mM CaCl_2_, 1 mM MgCl_2_, and 5 mM HEPES, pH 7.5) with additional 1 mM Na_2_SO_4_ or 300 µM Na-oxalate as indicated. HCO_3_^−^ solutions were continuously bubbled with 5% CO_2_/33 mM HCO_3_^−^ (pH 7.5). For unclamped Vm measurements, data were recorded with an OC-725C voltage clamp amplifier (Warner Instruments, Hamden, CT), filtered at 2–5 kHz, digitized at 10 kHz. For voltage clamp experiments, oocytes were clamped to −60 mV and then voltage rapidly shifted to −160 mV to +60 mV in 20 mV steps (70 ms sweeps, filtered at 1 kHz; HEKA Elektronik, Harvard Bioscience Inc.; https://www.heka.com/). The resulting linear IV-plots are then expressed as “slope conductance” in the appropriate non-CO_2_/HCO_3_^−^ solution (Chen et al., 2024). Full solution composition appears in Table 1. For ion-selective microelectrodes to monitor pH_i_, we used a liquid-ion-exchange (lix) resin (H^+^ ionophore I, mixture B; Fluka Chemical, Ronkonkoma, NY, United States). Intracellular pH microelectrodes had slopes of at least −54 mV/pH unit (Romero et al., 2000). Whenever possible, the slope of pH changes (pH-electrode and pH-sensor) within an individual trace was calculated from regions with linear change and overlapping range of absolute pH to minimize the effects of intrinsic and HCO_3_^−^-derived buffering on perceived rate of pH changes (Chesler, 1990; Rossano and Romero, 2017; Vaughan-Jones and Wu, 1990).

**TABLE 1:** Oocyte electrophysiology solutions.

Component	Oocyte solutions			
	ND96- HEPES	ND96- HCO_3_^−^/5%CO_2_	Sulfate HCO_3_^−^/5%CO_2_	Ox HCO_3_^−^/5%CO_2_
NaCl	96	63	61	62.4
KCl	2	2	2	2
CaCl_2_	1.8	1.8	1.8	1.8
MgCl_2_	1	1	1	1
NaHCO_3_	0	33	33	33
Na_2_SO_4_	0	0	1	0
Na_2_Ox	0	0	0	0.3
HEPES	5	5	5	5
pH	7.5	7.5	7.5	7.5

All values in mM, except pH (in pH units). pH set by addition of HCl and NaOH. HCO_3_^−^

solutions were continuously bubbled with 5% CO_2_ balanced with oxygen.

### 2.5 *Drosophila* husbandry

Flies were kept on standard medium in vials at 22°C, 12:12-h photoperiod, and 40% relative humidity. All lines were backcrossed into a w^1118^ (Bonini) background and this line served as controls.

### 2.6 Cell type-specific knockdown of CG5002 (Neat)

This was performed as described previously (Reynolds et al., 2024). To specifically knock down CG5002, we utilized the CapaR-GAL4 driver (Terhzaz et al., 2012) to restrict expression to the MT principal cells and crossed it VDRC lines KK100600 or GD10058 (Vienna, Vienna BioCenter Core Facility) containing a hairpin dsRNA sequence directed against CG5002.

### 2.7 Quantitative RT-PCR

Quantitative RT-PCR validation was performed as described elsewhere (Reynolds et al., 2024). mRNA was prepared from 7-day-old W^1118^ or experimental tubules using Qiagen RNAeasy column. Superscript II and an oligo-dT were used for reverse transcription. For each sample, 500 ng of cDNA were added to 12.5 μL of 2 μM SYBR green reaction mix (Finnzymes, GRI, Essex, United Kingdom) and 1 μL of 6.6 μM forward and reverse primers. An Opticon 2 thermocycler was set as follows: 95°C for 15 min followed by 35 cycles of 95°C for 30 s, 58°C for 30 s, and 72°C for 30 s. The ribosomal protein L32 (RPL32) gene was used as a standard in all experiments. For each condition, we used three independent samples; each PCR was repeated three independent times to verify results. Full primer sequences are listed in Table 2.

**TABLE 2:** Quantitative PCR primers.

Primer	Sequence (5′-3′)
RPL32 forward (control)	ATG CTA AGC TGT CGC ACA AAT G
RPL32 reverse (control)	GTT CGA TCC GTA ACC GAT GT
CG5002 forward	AAT TGG CAG TGC CCA GAT CA
CG5002 reverse	ACA CGG AAG GGT TGA TTC CC

### 2.8 *Ex vivo* cellular pH imaging of *Drosophila* MTs with a genetically-encoded pH indicator

Live epifluorescence imaging of pHerry (Rossano et al., 2017), a pseudo-ratiometric GEpHI consisting of a pH-sensitive superecliptic pHluorin (Miesenbock et al., 1998) (SepH; 488/510 nm ex./em.) and pH-insensitive mCherry (Shaner et al., 2004) (565/620 nm ex./em.) joined by a short peptide linker, was performed as previously described (Rossano and Romero, 2017). Briefly, UAS-pHerry-myr (encoding pHerry with an additional N-terminus myristylation sequence to aid in near-membrane targeting and nuclear exclusion) flies were crossed to CapaR-GAL4 flies to drive construct expression in the principal cells of the MT. Anterior MTs were extracted from 7d female flies and transferred to poly-l-lysine-coated slides. Preparations were moved to an inverted wide-field epifluorescent microscope with GFP (SEpH) and RFP (mCherry) filter sets (470/40 nm ex., 515 nm longpass em; 500 nm dichroic and 546/10 nm ex, 590 nm longpass em, 565 nm dichroic), a ×10/0.45 air objective, a monochromatic camera for live-image capture, and an in-line computer-controlled perfusion system. SEpH and mCherry images were collected in an interleaved fashion with 300 ms exposure for each channel and an additional 400 ms to allow for channel switching, yielding a total imaging rate of 1 Hz. MTs were continuously bathed in insect saline consisting of 121.5 mM NaCl, 20 mM KCl, 20 mM glucose, 8.6 mM HEPES, and 10.24 mM NaHCO_3_ with pH 6.8 set by HCl and NaOH. In NH_4_Cl pulse experiments NH_4_Cl was provided in equimolar exchange for NaCl. pH_i_ calibration was performed as previously described (Rossano et al., 2013; Rossano et al., 2017; Thomas et al., 1979) using modified solution consisting of 130 mM KCl, 20 mM Glucose, and 8.6 mM MES, HEPES, or TAPS as appropriate for desired pH range. pH was adjusted with HCl and NMDG and solution was supplemented with 10 μM nigericin in DMSO. Additional point calibrations were used at the end of some imaging experiments. When possible, control and experimental tubules were image simultaneously with the MTs positioned side-by-side to minimize the effects of variable perfusion rates between experiments. Rates of change in pH_i_ were compared in segments of traces within the same pH_i_ to minimize the influence of the pH-dependence of intracellular buffering (Chesler, 1990; Rossano and Romero, 2017; Vaughan-Jones and Wu, 1990). Imaging stacks were acquired in Intelligent Imaging Innovations Slidebook software, background corrected and exported for fluorescence quantification in ImageJ. Full compositions of solutions are described in Table 3.

**TABLE 3:** *Drosophila* experimental solutions.

Component	Insect saline (pH_i_ fluorescence experiments)						Insect saline (*ex vivo* crystallization)	
	HEPES	HCO_3_^−^/5%CO_2_	Sulfate	Ox	Calibration	NH_4_Cl	Standard	Ox
NaCl	131.5	121.5	119.5	119.6	0	81.5	121.5	101.5
KCl	20	20	20	20	130	20	20	20
Glucose	20	20	20	20	20	20	20	20
NaHCO_3_	0	10	10	10	0	10	10	10
NaH_2_PO_4_H_2_O	4.5	4.5	4.5	4.5	0	4.5	4.5	4.5
NH_4_Cl	0	0	0	0	0	40	0	0
Na_2_SO_4_	0	0	1	0	0	0	0	0
Na_2_Ox	0	0	0	1	0	0	0	10
HEPES	8.6	8.6	8.6	8.6	8.6 (pH 6.5–7.5)	8.6	8.6	8.6
MES	0	0	0	0	8.6 (pH < 6.5)	0	0	0
TAPS	0	0	0	0	8.6 (pH > 7.5)	0	0	0
pH	6.8	6.8	6.8	6.8	varies	6.8	6.8	6.8

All values are expressed in mM, except pH (in pH units). pH set by addition of HCl and NaOH in all solutions except calibration solution, in which case HCl and NMDG were used. HCO_3_^−^ solutions were continuously bubbled with 5% CO_2_ balanced with oxygen.

### 2.9 *Ex vivo* CaOx birefringence experiments

Adult flies (7d) were allowed to feed on standard food. Malpighian tubule (MT) from female flies were dissected in Schneider’s medium and transferred immediately to poly-l-lysine-coated slides with insect saline. MTs were allowed to settle for 10 min, at which point solution was swapped for saline supplemented with 10 mM Na-oxalate (equimolar exchange for NaCl).

### 2.10 CaOx crystal quantification

CaOx crystals were quantified using ImageJ software as previously (Landry et al., 2016). Images were taken at ×10 magnification with an air objective and an inline polarizer. 600 μm was measured from the terminal end of the respective MT. Once measured, brightness and contrast were adjusted, resulting in only birefringent crystals in the designated 600 μm of tubule being visible, and the remaining tubule artifacts were blacked out. A threshold was then set, resulting in binary coloration so that crystals could be counted. Crystals were analyzed using the Analyze Particles feature in ImageJ with the minimum desired size of stone detection specified at 2 μm^2^. Data generated included an accurate and specific crystal count. Data were then analyzed via one-way ANOVA with Bonferroni *post hoc* test (P < 0.05) or Student’s t-test (P < 0.05) using GraphPad Prism.

### 2.11 Data analysis and presentation

Data were compiled in GraphPad Prism and analyzed as indicated in individual data sets. Image analysis was performed in Intelligent Imaging Innovations Slidebook software as well as ImageJ. Curve fitting was performed in Microsoft Excel and GraphPad Prism. Representative micrographs were processed in Adobe Photoshop and figures were assembled with Adobe Illustrator.

### 2.12 Experimental solutions

Full Composition of all experimental solutions is described in Tables 1, 3.

### 2.13 Statistical methods

All statistical calculations were performed in GraphPad Prism. Comparison across two groups was performed by unpaired Student’s t-test. Comparisons more than two groups were performed by ANOVA with Bonferroni multiple comparisons correction. Sample sizes for experiments were determined by number of oocytes or number of extracted Malpighian tubules (one tubule per fly). Additional details are provided in descriptions of individual experiments.

## 3 Results

### 3.1 Which *Drosophila* Slc26 proteins transport oxalate in the Malpighian tubule ?

**FIGURE 2:**
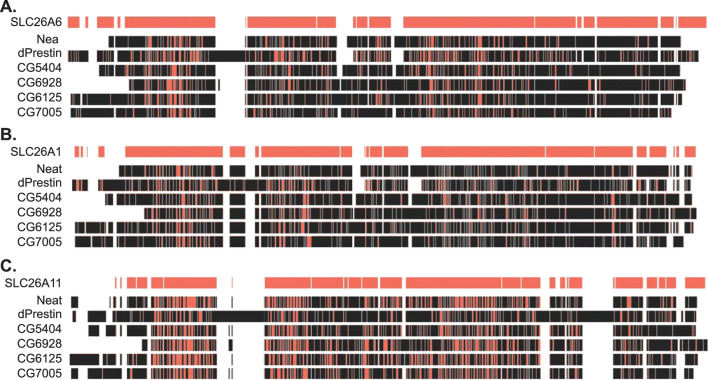
Amino acid sequence similarity between fly and human SLC26 proteins. **(A)** Amino acid sequence alignment of fly SLC26 proteins to human SLC26A6. **(B)** Amino acid sequence alignment of fly SLC26 proteins to human SLC26A1. **(C)** Amino acid sequence alignment of fly SLC26 proteins to human SLC26A11. Red indicates sequence match to human SLC26 sequences on the top line of each section. Alignment performed by Clustal Omega within the Benchling platform. Ascension numbers are as follows: SLC26A6, NM_134263; SLC26A1, AF297659; SLC26A11, AF345195; dPrestin, NM_140767; CG5002 (Neat), AY240021; CG5404, NM_142225; CG6125, AY240022; CG6928, AY240023; CG7005, NM_079766. The amino acid sequence alignment is in Supplementary Figure S1.

Previous work showed that apical protein dPrestin could transport oxalate into the Malpighian tubule lumen (Figure 1) (Hirata et al., 2012a). This dPrestin (Slc26a5/a6) is the only *Drosophila* Slc26 protein molecularly close to other known mammalian oxalate transporters (Hirata et al., 2012b) (Figures 2A,B). All others *Drosophila* Slc26 proteins, at the molecular sequence level, are most closely related to SLC26A11 (Figure 2C; Supplemental Figure S1), a Na^+^ independent SO_4_^2−^ transporter (Vincourt et al., 2003) and a Cl^−^/HCO_3_^−^ exchanger (Xu et al., 2011). SLC26A11/Slc26a11 proteins have not been reported to transport Ox^2−^.

**FIGURE 3:**
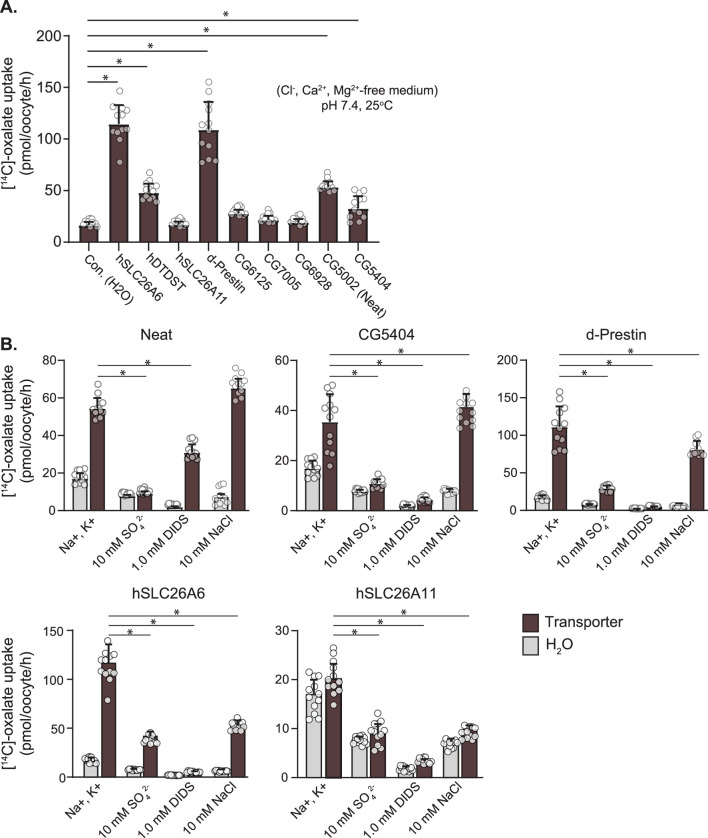
Ox^2−^ Uptake by *Drosophila* Slc26-transporters in *Xenopus* Oocytes. **(A)** ^14^C-labeled oxalate uptake in *Xenopus* oocytes expressing oxalate transporters and water injected control. **(B)** Response of ^14^C-labeled oxalate uptake in *Xenopus* oocytes expressing oxalate transporters and water injected controls to control solution, 10 mM SO_4_^2−^, 1 mM DIDS, and 10 mM NaCl. Circles indicate individual oocytes. Bars = mean + SD. N = 12 oocytes per group. * p < 0.05 by one-way ANOVA with Bonferroni multiple comparisons tests.

To first test for oxalate transport, we used ^14^C-oxalate uptake experiments with candidate transporters expressed in *Xenopus* oocytes (Figure 3A). Known human oxalate transporters (SLC26A6, SLC26A2/DTDST) and dPrestin were tested for oxalate uptake and compared to water-injected controls, SLC26A11, and *Drosophila* Slc26 clones [CG5002 (Neat), CG5404, CG6125, CG6928, CG7005]. As previously shown, SLC26A6 (114.60 ± 18.30 pmol/oocyte/h), DTDST (48.001 ± 8.66) and dPrestin (109.16 ± 26.80) all show significant oxalate uptake vs. control (16.63 ± 2.99, p < 0.00001) with SLC26A6 and dPrestin having the highest oxalate uptake without extracellular Cl^−^ (Figure 3A). SLC26A11 does not show any statistically significant oxalate uptake compared to the control (17.15 ± 2.78, p = 1). Of the *Drosophila* Slc26 proteins, only Neat (53.43 ± 5.49, p < 0.00001) and CG5404 (32.74 ± 11.83, p < 0.05) showed significant oxalate uptake in Cl^−^ free solution. CG6125 (27.69 ± 3.69, p = 0.98), CG6928 (19.27 ± 3.17, p = 1) and CG7005 (21.62 ± 4.00, p = 1) did not differ from controls (16.63 ± 2.99).

Within these same uptake experiments, cis-inhibition by 10 mM SO_4_^2−^ and 1 mM DIDS were evaluated (Figure 3B). As expected from the previously published activity of dPrestin (111.14 ± 27.28 pmol\oocyte/h), both DIDS (29.41 ± 3.64) and SO_4_^2−^ (4.69 ± 0.73) cis-inhibit dPrestin oxalate uptake to water injected control levels (16.94 ± 3.04; SO_4_^2−^, 7.74 ± 0.65; DIDS, 1.91 ± 0,38). A similar cis-inhibition pattern was found comparing CG5404 to control Ox^2−^ uptakes. For Neat (54.40 ± 5.59), cis-addition (bath) of 10 mM SO_4_^2−^ (9.03 ± 1.22) fully blocked Ox^2−^ uptake (Figure 3B, left). However, 1 mM DIDS only reduced Ox^2−^ uptake by half (30.92 ± 4.29).

Many SLC26 protein family members transport chloride, and thus we evaluated if cis application of Cl^−^ in previously chloride-free solutions could inhibit Ox^2−^ uptake within the same experiments (Figure 3B, right sides of panels). Both Neat (54.40 ± 5.59) and CG5404 (32.74 ± 11.83) displayed no significant cis inhibition of Ox^2−^ uptake by Cl^−^ (66.29 ± 5.18 and 37.91 ± 4.64 respectively). Chloride inhibited Ox^2−^ uptake by dPrestin by approximately half (111.14 ± 27.28 to 68.52 ± 9.45). Similarly robust inhibition of oxalate uptake by cis application of Cl^−^ was observed in SLC26A6 (114.60 ± 18.30 to 52.82 ± 5.07). Taken together, these data support our ability to detect the effects of extracellular Cl^−^ on Ox^2−^ uptake and reveal Neat does not function as an Ox^2−^/Cl^−^ exchanger.

### 3.2 Which *Drosophila* Slc26 mRNAs exist in the MT?

The next consideration was which *Drosophila* Slc26 oxalate transporter mRNAs or proteins have expression in MTs. Searching FlyAtlas2 (flyatlas2.org), we found that Neat was 175 ± 21 FPKM (adult male) and 191 ± 27 FPKM (adult female) while CG5404 is 16 ± 2.5 FPKM (male) and 15 ± 4.0 FPKM (female). These data indicate that Neat mRNA expression is ∼10-fold higher than cg5404, and that oxalate uptake is ∼2-fold higher. The combined data indicate that Neat is the *Drosophila* Slc26 transporter of most interest for a candidate basolateral Ox^2−^ transporter in the Malpighian tubule. CG5404 expression is increased relative to Neat in hindgut (140 vs 18 FPKM in adult females).

### 3.3 Electrophysiology of Neat function – *Xenopus* oocytes

**FIGURE 4:**
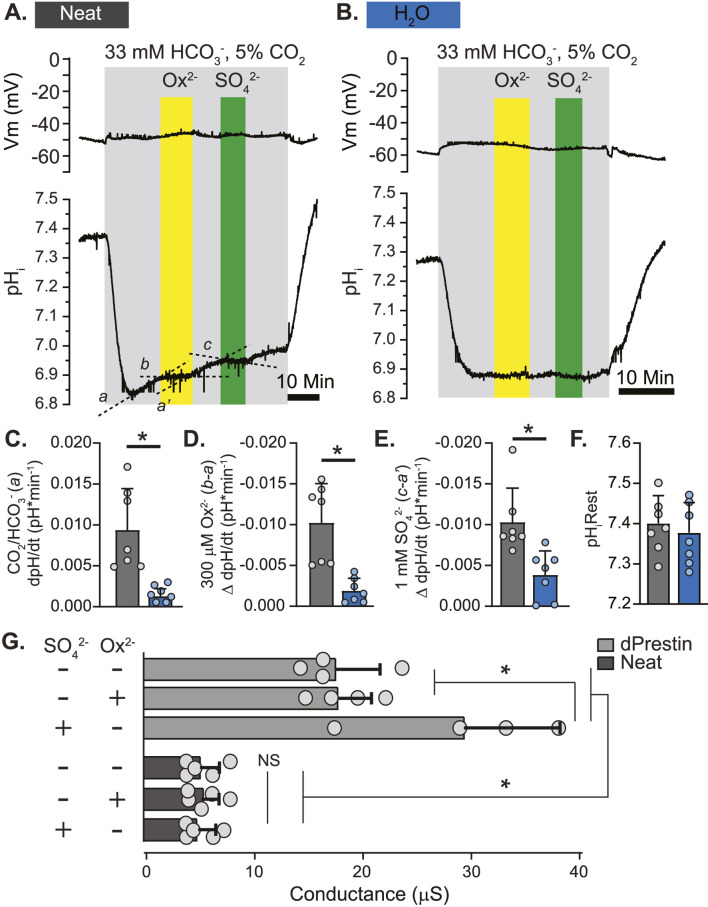
Putative *Drosophila* Ox^2−^ Transporter “Neat” Displays Electroneutral HCO_3_^−^/Ox^2−^ and HCO_3_^−^/SO_4_^2−^ Exchange in *Xenopus* Oocytes. **(A, B)** simultaneous un-clamped membrane voltage (Vm; voltage electrode) and pH_i_ (intracellular pH-sensitive electrode) responses to extracellular Ox^2−^ (300 μM) and SO_4_^2−^ (1 mM) in *Xenopus* oocytes expressing Neat **(A)** and control (H_2_O; **(B)**. **(C–E)** Rate of change of pH_i_ in response to CO_2_/HCO_3_^−^ solution **(C)**, Ox^2−^ **(D)**, and SO_4_^2−^ **(E)**. Slopes derived from a, b, a’, and c in **(A)**. The same regions were used to calculate slopes in control (H_2_O) oocytes, but labels are omitted from B for clarity. Circles = individual oocytes. Bars = Mean + SD. * = p ≤ 0.05 by two-way unpaired Student’s t-test. **(F)**. Initial pH_i_ prior to 5%CO_2_/33 mM HCO_3_^−^ solution (pH 7.5). Circles = individual oocytes. Bars = Mean + SD. **(G)** Basal and oxalate/sulfate-induced conductance measurements obtained by voltage clamp in oocytes expressing dPrestin and Neat. Circles = individual oocytes. Bars = Mean + SD. *p < 0.01 by one-way ANOVA with Bonferroni *post hoc* test.

With Neat identified as the likely oxalate transporter of interest we further characterized its transport of Ox^2−^, HCO_3_^−^, and SO_4_^2−^ in a *Xenopus* oocyte heterologous expression system (Figure 4). Neat demonstrated electroneutral HCO_3_^−^ influx in the presence of HCO_3_^−^/CO_2_ buffered saline (dpH/dt 0.0094 ± 0.0047 pH units per minute as compared to 0.0013 ± 0.0003 in water injected control oocytes; Figures 4A–C). This influx of HCO_3_^−^ was stopped by the addition of 300 µM Ox^2−^ (ΔdpH/dt: −0.010 ± 0.004; Figure 4D) or 1 mM SO_4_^2−^ (ΔdpH/dt: −0.010 ± 0.004; Figure 4E). Each of the substrates produced significant changes in dpH/dt compared to those observed in water injected oocytes (p < 0.005 by Student’s unpaired t-test in all cases). Expression of Neat did not significantly alter resting pH_i_ (Figure 4F). These electrophysiology data suggest Neat mediates electroneutral HCO_3_^−^/Ox^2−^ exchange and HCO_3_^−^/SO_4_^2−^ exchange. Voltage clamp experiments revealed that dPrestin elicited conductance changes with 5 mM SO_4_^2−^ as previously reported (Hirata et al., 2012b). However, neither Ox^2−^ nor SO_4_^2−^ elicited a conductance change from baseline in Neat. Additionally, the overall Neat-oocyte conductance (5.94 ± 2.03 µS) was about 25% of baseline dPrestin conductance (19.44 ± 4.25 µS; Figure 4G). These data further support the conclusion that ion transport via Neat is electroneutral.

### 3.4 Neat function in *Drosophila* MTs

**FIGURE 5:**
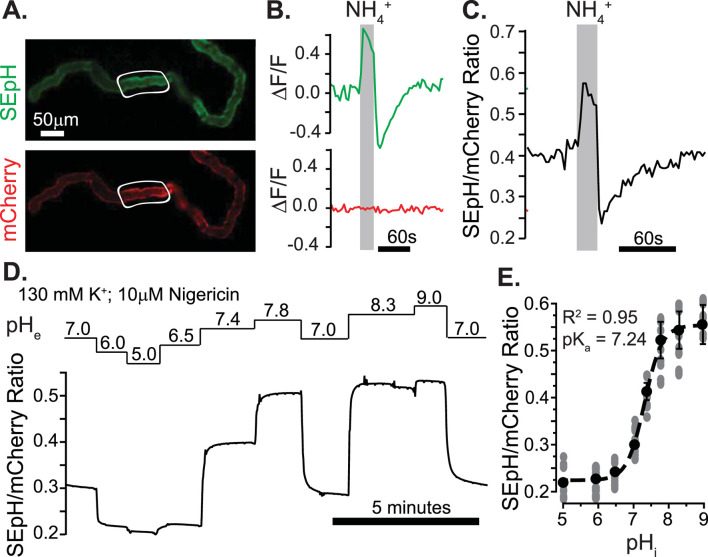
*Ex vivo* quantification of intracellular pH in transitional segment MT principal cells with a pseudo-ratiometric genetically-encoded pH Indicator. **(A)** Fluorescent micrograph of dissected MT from UAS-pHerry-myr; CapaR-GAL4 female fly displaying pH-sensitive (SEpH; 488 nm ex./510 nm em) and pH-insensitive (mCherry; 565 nm ex./630 nm em) components of pHerry. **(B)** Relative fluorescence changes in SEpH and mCherry in response to Intracellular alkalinization and acid loading by bath-applied 40 mM NH_4_Cl pulse. Traces derived from background corrected ROIs in **(A)** (white outlines). **(C)** Ratio of SepH to mCherry signal from **(B)**. **(D)** Representative trace of normalized SEpH/mCherry ratio in response to high K^+^/Nigericin calibration with imposed extracellular pH (pH_e_) values. **(E)** Calibration curve of pHerry ratio derived from Boltzmann fit of data from **(D)**. Circles = individual principal cells, data set derived from 8 MTs. In all cases SepH and mCherry frames were captured at 300 ms each with 400 ms allotted for filter switching, yielding an imaging rate of 1 Hz.

The fact that Neat mediates exchange of acid equivalents raised the possibility that we could investigate Neat activity in *Drosophila* MTs *ex vivo* through the use of genetically encoded pH indicators targeted to the cytosolic surface of the MT principal cell membrane. To this end, we used the GAL4/UAS binary expression system to express pHerry (Rossano et al., 2017), a pseudo-ratiometric genetically encoded pH indicator consisting of a pH-sensitive GFP variant (SEpH) and a pH insensitive RFP variant (mCherry) in the principal cells of adult *Drosophila* MTs and calibrated the biosensor to monitor pH_i_ in real time through live epifluorescent imaging as previously described (Rossano and Romero, 2017). Both the GFP and RFP components of pHerry were easily visualized in extracted MTs (Figure 5A), and direct manipulation of pH_i_ with NH_4_Cl pulses confirmed the expected pH sensitivity of the SEpH component and the pH insensitivity of the mCherry component (Figures 5B,C). We used the high potassium/nigericin technique (Thomas et al., 1979) to calibrate the GFP/RFP ratio as a function of pH_i_ (Figures 5D,E). Boltzmann fit to these data yielded R^2^ = 0.95 and pK_a_ = 7.24, which is in agreement with previously published *ex vivo* and *in situ* calibration of superecliptic pHluorin-based pH Indicators in *Drosophila* (Rossano et al., 2013; Rossano et al., 2017).

**FIGURE 6:**
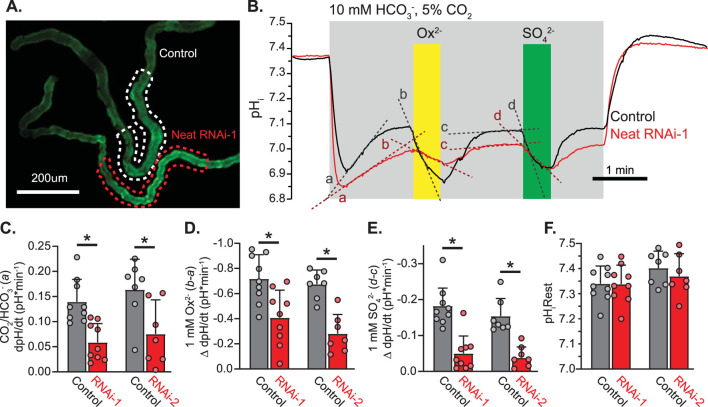
*Drosophila* CG5002 (Neat) contributes to basolateral HCO_3_^−^/Ox^2−^ and HCO_3_^−^/SO_4_^2−^ exchange in transitional segment MTs *ex vivo* **(A)**. Representative fluorescent micrograph of SEpH component of pHerry in MT principal cells from control (UAS-pHerry-myr; CapaR-GAL4) and Neat knockdown (UAS-pHerry-myr; UAS-RNAi_KK100600_; CapaR-GAL4) flies. **(B)** Representative traces of transitional segment principal cell pH_i_ derived from calibrated pHerry fluorescent ratio demonstrating pH response to bath-applied CO_2_/HCO_3_^−^, Ox^2−^, and SO_4_^2−^ solutions. Traces correspond to dashed ROIs in A and represent the average of all principal cells within the transitional segment. Note recovery of pH_i_ following an initial acidification in CO_2_/HCO_3_^−^ solution is reversed by both oxalate and sulfate, although to a lesser extent when Neat is knocked down. **(C–E)** Change in the rate of pH_i_ change in response to 5%CO_2_/10 mM HCO_3_^−^ solution (pH 6.8) **(C)**, Ox^2−^ **(D)**, and SO_4_^2−^ **(E)**. RNAi-1 and RNAi-2 denote two separate RNA-i constructs targeting Neat (KK100600 and GD10058). Each experiment conducted with a control and Neat RNA-i MT in the same field of view as in **(A)**. Slopes derived from a, b, c, and d in **(B)**. Circles = single MTs. Bars = Mean + SD. * = p ≤ 0.05 by two-way unpaired Student’s t-test. **(F)** Initial pH_i_ prior to 5%CO_2_/10 mM HCO_3_^−^ solution (pH 6.8). Circles = individual MTs. Bars = Mean + SD.

We next combined live imaging of pH_i_ with the robust RNAi toolkit available in *Drosophila* to evaluate changes in pH_i_ of MT principal cells attributable to HCO_3_^−^, SO_4_^2−^, and Ox^2−^ in the presence and absence of Neat knockdown with two separate RNAi constructs (KK100600 and GD10058 from the Vienna stock center). When CapaR-GAL4 was used to drive RNAi expression in MT principal cells quantitative PCR revealed knockdown of transcript level to 34% ± 0.5% and 40% ± 0.5% of control level in RNAi-1 (KK100600) and RNAi-2 (GD10058), respectively. Transcript levels were normalized to RPL32 in all cases and data were pooled across three runs from each genotype in RNA extracted from adult *Drosophila* MTs. Freshly extracted MTs expressing membrane-targeted pHerry were mounted on cover glass and perfused with insect saline buffered either with HEPES or HCO_3_^−^/CO_2_ with additional oxalate and sulfate to monitor changes in pH_i_ in response to basolateral addition of HCO_3_^−^, Ox^2−^, and SO_4_^2−^ (Figures 6A,B). Switching to HCO_3_^−^ buffered saline produced an initial fall in pH_i_ followed by a gradual alkalinization mediated by HCO_3_^−^ influx. This alkalinization was significantly slowed when Neat was knocked down with either RNAi construct as compared to that seen in control MTs (ΔdpH/dt: +0.14 ± 0.04 vs. +0.06 ± 0.04 in RNAi-1 experiments and +0.17 ± 0.06 vs. +0.07 ± 0.06 in RNAi-2 experiments, p < 0.01 in both cases by Students unpaired t-test; Figure 6C). This alkalinization was quickly reversed with the addition of 1 mM basolateral oxalate as well as 1 mM basolateral sulfate. In each case the change in dpH/dt with the addition of oxalate (−0.72 ± 0.18 vs. −0.41 ± 0.21 in RNAi-1 and -0.67 ± 0.10 vs. −0.28 ± 0.14 in RNAi-2) and sulfate (−0.19 ± 0.06 vs. −0.05 ± 0.05 in RNAi-1 and -0.15 ± 0.05 vs. −0.04 ± 0.03 in RNAi-2) was significantly reduced by knockdown of Neat (Figures 6D,E). Knockdown of Neat did not change resting pH_i_ in the principal cells of the transitional segment of MTs (Figure 6F). Taken together with our prior oocyte expression experiments these data strongly support the conclusion that Neat mediates significant HCO_3_^−^/Ox^2−^ and HCO_3_^−^/SO_4_^2−^ exchange on the basolateral membrane of *Drosophila* MT principal cells of the transitional segment.

### 3.5 Neat and CaOx crystallization in *Drosophila* MTs

**FIGURE 7:**
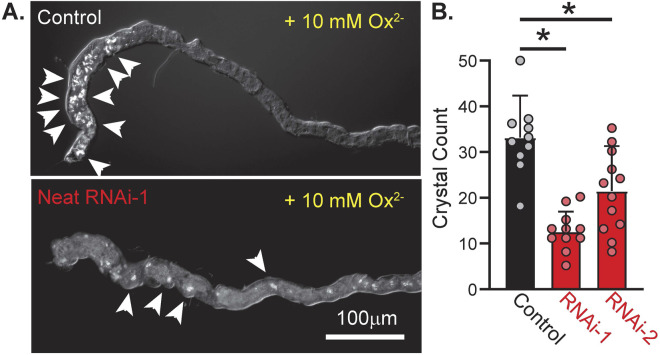
Knockdown of CG5002 (Neat) reduces accumulation of oxalate crystals in MT lumen during basolateral application of oxalate solution. **(A)** Representative polarized light images of *ex vivo* MTs from control (UAS-pHerry-myr; CapaR-GAL4) and Neat knockdown (UAS-pHerry-myr; UAS-RNAi_KK100600_; CapaR-GAL4) flies after 60 min exposure to bath-applied 10 mM Ox^2−^ solution. Arrowheads denote prominent crystal formation. **(B)** Crystal count from lumen of MTs following 60-min Ox^2−^ exposure. Circles indicate individual MTs. Bars = mean + SD. * = p ≤ 0.05 by one-way ANOVA with Bonferroni multiple comparisons tests.

We hypothesized that if Neat was a significant mediator of basolateral Ox^2−^ transport in the *Drosophila* MT then knockdown of this transporter should significantly impair transepithelial transport of oxalate and formation of CaOx crystals in the MT lumen. Previous work has demonstrated that knockdown of the apical oxalate transporter in the MT significantly reduces luminal CaOx crystal formation when Ox^2−^ solution is perfused across extracted *Drosophila* MTs (Landry et al., 2016). To test if this was the case for the putative basolateral Ox^2−^ transporter, we assessed CaOx crystal burden in extracted MTs from control and Neat knockdown flies via polarizing birefringence. 60-min bath application of insect saline containing 10 mM Ox^2−^ was sufficient to induce prominent crystal formation (Figure 7A). CaOx crystal count was significantly reduced when Neat was knocked down by both RNAi-1 and RNAi-2 constructs (12.64 ± 4.14 and 21.5 ± 9.39 for each construct respectively vs. 33.20 ± 8.67 in control, p < 0.0001 by one-way ANOVA with Bonferroni multiple comparisons correction; Figure 7B). These data suggest Neat significantly contributes to transepithelial Ox^2−^ movement, and this contribution is of similar magnitude to that of the previously described apical Ox^2−^ transporter dPrestin (Landry et al., 2016).

## 4 Discussion

*Drosophila melanogaster* has been widely used as a credible model in exploring the pathophysiology of CaOx nephrolithiasis (Akouris et al., 2022; Al et al., 2020; Ali et al., 2018; Branco et al., 2021; Chen et al., 2012; Chen et al., 2011; Chi et al., 2015; Chung et al., 2016; El-Salam et al., 2018; El-Salam et al., 2017; Farkaš et al., 2016; Han et al., 2019; Hirata, et al., 2012a; Landry et al., 2019; Landry et al., 2016; Lu et al., 2024; Miller et al., 2013; Reynolds et al., 2024; Sun et al., 2022; Yang et al., 2018). Our previous experiments illustrated that Slc26a6 ortholog dPrestin mediated oxalate export on apical membrane of *Drosophila* renal tubule. To better understand how oxalate crosses into the MT lumen and affects CaOx crystallization in MTs, we undertook the present work to characterize another *Drosophila* Slc26 oxalate transporter. These studies focused on *Drosophila* GC5002 (Neat), and we found that Neat contributes to Ox^2−^ transepithelial movement in MTs via electroneutral HCO_3_^−^/Ox^2−^ and HCO_3_^−^/SO_4_^2−^ exchange. Sequence homology suggested several previously uncharacterized *Drosophila* oxalate transporters within the Slc26 family, but Ox^2−^ uptake experiments only revealed significant oxalate transport via Neat and CG5404 (Figure 3A). Ox^2−^ uptake by Neat was almost completely inhibited by SO_4_^2−^ and partly inhibited by DIDS in Cl^−^ free solution (Figure 3B). Surprisingly, application of extracellular chloride did not inhibit Ox^2−^ uptake by Neat, consistent with the interpretation that Neat does not function as an Ox^2−^/Cl^−^ exchanger. Available expression data indicated that CG5404 was minimally expressed in the MT while Neat was highly expressed, suggesting it was the most likely candidate to function as the basolateral Ox^2−^ transporter contributing to transepithelial Ox^2−^ movement in concert with apical dPrestin.

Cis-application of SO_4_^2−^ blocked Ox^2−^ uptake via Neat (Figure 3B), and electrophysiological recordings in *Xenopus* oocytes revealed both electroneutral HCO_3_^−^/Ox^2−^ and HCO_3_^−^/SO_4_^2−^ exchange (Figures 4A,G). Taken together, these data suggest both Ox^2−^ and SO_4_^2−^ share a binding site on Neat and ion exchange occurs via movement of base equivalents. An alternative explanation is that Neat engages in electroneutral exchange of HCO_3_^−^ for another ion, and this activity is competitively inhibited by both Ox^2−^ and SO_4_^2−^. The observation that Neat does not reduce Ox^2−^ uptake in response to Cl^−^ (Figure 3B) makes this unlikely, but we cannot completely rule out this possibility as Neat mediates electroneutral HCO_3_^−^ influx in both oocytes and MTs even in the absence of Ox^2−^ and SO_4_^2−^ (slope “a” in Figures 3A,C and slope “a” in Figures 6B,C respectively). Future studies are needed to determine if this HCO_3_^−^ influx is balanced by movement of Cl^−^, OH^−^, or metabolic substrates such as formate^−^ (also transported by some Slc26 proteins).

We validated tools for measuring intracellular pH at a cellular scale within the principal cells that comprise the *Drosophila* MT (Rossano and Romero, 2017) (Figure 5). We combined these quantifiable live imaging tools with multiple RNAi constructs and tissue specific transgene expression to knockdown Neat in the principal cells of the MT while monitoring the effect on pH_i_ changes elicited by basolateral perfusion of oxalate and sulfate in HCO_3_^−^/CO_2_ buffered saline (Figure 6). These experiments were in agreement with the prior heterologous expression systems data as they revealed HCO_3_^−^/Ox^2−^ and HCO_3_^−^/SO_4_^2−^ exchange on the basolateral membrane of the MT. These transport modalities were significantly attributable to Neat as knockdown with two separate RNAi constructs, validated to reduce transcript levels to approximately 34% and 40% of control lines, significantly reduced base equivalent movement in the presence of both Ox^2−^ and SO_4_^2−^ (Figures 6B–E). Although we cannot confirm the exact subcellular distribution of Neat within MT principal cells or the extent of Neat protein knockdown, the basolateral membrane is the most likely location as all of the changes in pH_i_ were elicited by basolateral bath perfusion of substrates. Perfusion of the apical surface was likely minimal as the tubules are pinched shut when mounted on cover glass. Prior work (as well as data presented here; Figure 7) has demonstrated that genetic manipulation of Ox^2−^ transport is sufficient to alter calcium oxalate crystal formation in the Malpighian tubule lumen (Landry et al., 2016). These results are inconsistent with persistent apical perfusion by bath application of solutions. Simultaneous availability of soluble oxalate to the apical and basolateral surfaces would make crystal formation a function of transepithelial Ca^2+^, not Ox^2−^, transport. Such transport has not been observed or hypothesized for either Neat or dPrestin.

Characterization of the basolateral Ox^2−^ transporter within the MT is an important step in understanding *Drosophila* secretory biology, but we further characterized the role of Neat in contributing to a fly model of renal nephrolithiasis to demonstrate that modulation of Neat (and its mammalian functional orthologs) may present a novel target for therapeutics in oxalate kidney stone pathology. Knockdown of Neat reduced luminal CaOx crystal formation after bath application of 10 mM Na-oxalate (Figure 7). Mutations in human SLC26A1 which decrease transporter activity and impair membrane trafficking are associated with calcium oxalate nephrolithiasis. This is likely due to expression of SLC26A1 in the distal ileum, cecum, and proximal colon in humans, as disruption of basolateral Ox^2−^ transporter in those organs leads to retention of oxalate, hyperoxaluria, and ultimately hyperoxaluria (Gee et al., 2016). The fly MT represents a much simpler and experimentally tractable system, and our experiments demonstrate that it recapitulates key aspects of oxalate transepithelial movement. Future studies will need to account for diverse expression patterns of transporters across different tissues. Additional studies in flies using dietary oxalate may resolve the contribution of Neat to whole animal oxalate processing, but such experiments were beyond the scope of this study as the full tissue expression profile and subcellular localization of Neat remains to be characterized.

## 5 Conclusion

**FIGURE 8:**
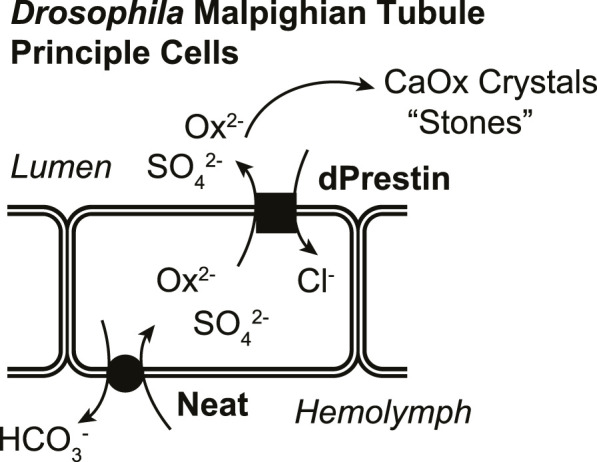
Proposed model of basolateral and apical Ox^2−^ movement across MT principal cells.

In conclusion, Neat contributes to sulfate and oxalate transport at the *Drosophila* MT as it mediates transepithelial exchange of HCO_3_^−^/Ox^2−^ and HCO_3_^−^/SO_4_^2−^. Together with dPrestin on apical membrane, Neat forms the basolateral component of transepithelial oxalate movement (Figure 8). The studies presented here demonstrate the power of the fly MT in modeling the physiology of nephrolithiasis as we were able to characterize the transport modalities of Neat within an *ex vivo* cellular system using live imaging of GEpHis, cell-specific RNAi, and a whole organ functional assay of oxalate crystal formation. These data were in agreement with Neat activity in heterologous expression systems. This research not only fills a critical gap in our understanding of oxalate transport within the *Drosophila* MT but also lays the groundwork for further exploration into the genetic and molecular mechanisms underlying human nephrolithiasis and potential therapeutic targets.

## Data Availability

The raw data supporting the conclusions of this article will be made available by the authors, without undue reservation.
