# Supplementary material for: Ex vivo quantification of intracellular pH in Drosophila Malpighian tubule reveals basolateral HCO3 −/oxalate exchange through a novel oxalate transporter “Neat”
Source: Front Physiol. 2025 Apr 28;16:1468451. doi: 10.3389/fphys.2025.1468451 (PMC12066474; doi:10.3389/fphys.2025.1468451)
Supplement: Supplementary file 1 [file Image1.pdf]

Supplemental Figure 1: Sequence alignment of all SLC26 members and candidates included in manuscript

```
CG5404          ----------------------------MYPQLETEAEALEKAT--------ARALLPGA
CG6125          -----MRWPRKHT-------------FGLWPKRNGRNRSSGEATGGQPQATVATIYDPGC
CG6928          ------------------------------------------------------------
SLC26A11        ------------------------------------------------------------
Neat            --------------------------------------------------MRADEDNLYR
CG7005          ---MKSNPASDHVYFNDGFKCSTISISTNLTEPNGSSNSVGSQG-----SKEFILTEDGK
dPrestin        MP-SDKESPE--------------------TKPLNNNNTNG--NGGSVP-SKPKIQPKYS
SLC26a6         MGLADASGPR---------------------------DTQA--LLSATQ-AMDLRRRDYH
SLC26A1         ---------------------------------------------------------MDE

CG5404          ISKPAS-----------------EAGKAPRMNKWKRRL-------------HRHVPVFQW
CG6125          EEKEKYTAQNGDDYYGQAHTVDLDVASKYSKPKEAHWL-------------VRRIFFLSW
CG6928          -------------------------------MSLRKWG-------------YRLLPGLKW
SLC26A11        -MPSSV------TALGQARSSG-PGMAPSACCCSPAAL-------------QRRLPILAW
Neat            EQLPNV--------------ST-LIRDGGRKLCRPSTV-------------TNKFPILKW
CG7005          KVKPTV------STLD--CTRS-WLQDCQRRTFNRKTL-------------HKRLPILGW
dPrestin        IHRDVLTHEVVIKQTGYAARDKSIPSSL--RNCWRSWN--------FFALFTGVIPILQW
SLC26a6         MERPLLNQEHLEE----LGRWGSAPR----THQWRTWLQCSR--ARAYALLLQHLPVLVW
SLC26A1         SPEPLQQGRGPVP----VRRQRPAPRGLREMLKARLWCSCSCSVLCVRALVQDLLPATRW

CG5404          LPLYS-TEWGIDDFIAGITLGLTIIPESMACALLAGLPARYGLCSAFIGPLIYMVFGSID
CG6125          ITSYD-REQAFADLIAGITLGLTIIPQSIAYAALAGLSSEYGLYSAFIGSIIYVFFGTIP
CG6928          LHGYT-GQDAVADLIAGVTVGLTVLPQGLAYATLAGLEPQYGLYSAFVGGIIYAMLGSCR
SLC26A11        LPSYS-LQWLKMDFVAGLSVGLTAIPQALAYAEVAGLPPQYGLYSAFMGCFVYFFLGTSR
Neat            LPRYR-LEYIMQDFIAGFTVGLTTIPQAIAYGVVAGLEPQYGLYSAFMGCFTYIVFGSCK
CG7005          LPKYN-SQDAVGDLVAGITVGLTVIPQALAYAGIAGLPVAYGLYASFVGCFVYIFLGSCK
dPrestin        LPQYSPRRDLPGDIIAGFTVAIMNIPHGMAYGILAGVSAGNGLYMAVFPVLAYMFLGTSK
SLC26a6         LPRYPVRDWLLGDLLSGLSVAIMQLPQGLAYALLAGLPPVFGLYSSFYPVFIYFLFGTSR
SLC26A1         LRQYRPREYLAGDVMSGLVIGIILVPQAIAYSLLAGLQPIYSLYTSFFANLIYFLMGTSR

CG5404          KVIIGPTSLVALVSVQFTV---GR------------------------------------
CG6125          QVSIGPTSLMAILTLQFCA---DK------------------------------------
CG6928          QVTIGPTALLALMTSRHTGFGLGS------------------------------------
SLC26A11        DVTLGPTAIMSLLVSFYT----FH------------------------------------
Neat            DVTIATTAIMALMVNQYA----TI------------------------------------
CG7005          DVPMGPSAIVALLTYQAA----QG------------------------------------
dPrestin        HISIGTFAVASMMTAKVVDTYANVDDHHQILPINAFGLQSNGTATASPLLL-INSSALAD
SLC26a6         HISVGTFAVMSVMVGSVTESLAPQA---------------------------LNDSMI--
SLC26A1         HVSVGIFSLLCLMVGQVVDRELQLA---------GFDPSQDGLQPGANSSTLNGSAAM--

CG5404          -----------------------PIEFAFLLTFLSGIVQIIMGTMRMGFIFEFISMPVIK
CG6125          -----------------------PVQVVIVLAFLAGLVELAMGVFQLGFIVSFIPAPVTK
CG6928          -----------------------GPAYAILLCLISGVVELGMAVLKLGALVDLISLPVTV
SLC26A11        -----------------------EPAYAVLLAFLSGCIQLAMGVLRLGFLLDFISYPVIK
Neat            -----------------------SPDYAVLVCFLAGCIVLLLGLLNMGVLVRFISIPVIT
CG7005          -----------------------SWQKSVLLCLLSGIVELLMGLFGLGFLIDFVSGPVSS
dPrestin        SVSSTTISPFQLLNSTLNADPITKIEVATSLALTVGIVNLLMAFLRLGTLSSLLSEPLVN
SLC26a6         ---------------NETARDAARVQVASTLSVLVGLFQVGLGLIHFGFVVTYLSEPLVR
SLC26A1         ---------------LDCGRDCYAIRVATALTLMTGLYQVLMGVLRLGFVSAYLSQPLLD

CG5404          AFSSATAILVIESQLKVLLGIKYL-VAGL---MNSVGMLSSRIEESNMADLIMG-VCAIV
CG6125          AFTSGTALIVVFAQIKNLLGVRIKG-------FPSIGDFFT---NIRPTDAAMG-ISCMV
CG6928          GFTSATAVIIGTSQLKGLLGLRGGSGSDF---INTMRSVFGNLHKVRTGDFTLG-LTSII
SLC26A11        GFTSAAAVTIGFGQIKNLLGLQNI-PRPF---FLQVYHTFLRIAETRVGDAVLG-LVCML
Neat            GFTMAAATTIGSAQINNIVGLTSP-SNDL---LPAWKNFFTHLTSIRLWDALLG-VSSLV
CG7005          GFTSAVSLIILTSQIQSVLGITAK-GNTF---VEIWTQVFHNIEHTRAGDTVLG-LTCIV
dPrestin        GFTTAAACHVVTAQLKDVLGISVPRHKGAFKIIYTVIDVIKGVPQTNLVNFGFCMAVIAF
SLC26a6         GYTTAAAVQVFVSQLKYVFGLHLSSHSGPLSLIYTVLEVCWKLPQSKVGTVVTA-AVAGV
SLC26A1         GFAMGASVTILTSQLKHLLGVRIPRHQGPGMVVLTWLSLLRGAGQANVCDVVTS-TVCLA
```

```
CG5404        FLLLLELLDRVA--------NNEKRNKILRIFCRYLSTSRNTLIVLIAAIVSFIWIQ--K
CG6125        VLLSLRLLSQVN-----FKQDTPVTRRLKKILWY-ISISRNALVVFFTGLLVFIWVKKSS
CG6928        VLLLLRKLKDVKLDGRI--RNLRTQQLVSGSIWV-IGTGRNALVVLVTSVLAYSTCE--Q
SLC26A11      LLLVLKLMRDHV--PPVHPEMPPGVRLSRGLVWA-ATTARNALVVSFAALVAYSFEV---
Neat          FLLLMTRVKDI--------------KWGNRIFWKYLGLSRNALAVIFGTFLAYILSR---
CG7005        ILLLMRSLSSCRIGPVDEKECSSFQRAVNKILWI-VGTARNAILVVVCCIMGYMLHT--E
dPrestin      MMICNEILKPRLSK-----------------KCR-FPLPAELIMVIGGTLISKWFNL---
SLC26a6       VLVVVKLLNDKLQQ-----------------QLP-MPIPGELLTLIGATGISYGMGL---
SLC26A1       VLLAAKELSDRYRH-----------------RLR-VPLPTELLVIVVATLVSHFGQL---


CG5404        CGQVPYALSKNALSTLPNFTVPSFHIETA-----ERNYSIWEVLKELNIGIIVIPIVGIL
CG6125        IEAVPFALSSKVSSAMPTIKLPPFAFEYQ-----NRTYVFTDILHELGSGIVVVPIVAVL
CG6928        MESCPFILTGKVKSGLPNVSLPKFETTILDRNGTEIRQNFEQMLSELGPSMLILPIIAVL
SLC26A11      TGYQPFILTGETAEGLPPVRIPPFSVTTA-----NGTISFTEMVQDMGAGLAVVPLMGLL
Neat          DGNQPFRVTGNITAGVPPFRLPPFSTTVD-----GEYVSFGEMISTVGASLGSIPLISIL
CG7005        EHGAPFRVVGEIPPGLPSIQLPPTSLTANETS-NGVAEGFVEMVHSMGSGLVVIPLISLM
dPrestin      YVDYNVNPVGKIPSGLPEPVLPRLDLV-----------------PKVAVDSIAIAIVTYS
SLC26a6       KHRFEVDVVGNIPAGLVPPVAPNTQLF-----------------SKLVGSAFTIAVVGFA
SLC26A1       HKRFGSSVAGDIPTGFMPPQVPEPRLM-----------------QRVALDAVALALVAAA

CG5404        TNISIGKLTPK---GLVDTNQELLTVGLCNMFGSCVQAMPSSGAFTRYAISTACGLRTPM
CG6125        ANVAIAKAFVK--DGNLDASQEMLTLGLCNIAGSFFSAMPTCGAFTRSAVSQASGVRTPM
CG6928        GNVAISKAFGG---AGLSPTRELVALSMSNICGAFCSSMPVTGSFSRSAVNHASGVRTPL
SLC26A11      ESIAVAKAFASQNNYRIDANQELLAIGLTNMLGSLVSSYPVTGSFGRTAVNAQSGVCTPA
Neat          EIVAISKAFSK--GKIVDASQEMVALGMCNIMGSFVLSMPVTGSFTRTAVNNASGVKTPL
CG7005        ENIAICKAFAN--GKPVDASQELIAIGTANIFNSFVQAFPGTGALSRGAVNNASGVRTPL
dPrestin      IIMSMGLTFAKKHGYEVRPNQELFAMGIGNMVGGCFSCIPMACSLSRSVIQDQTGGVSQI
SLC26a6       IAISLGKIFALRHGYRVDSNQELVALGLSNLIGGIFQCFPVSCSMSRSLVQESTGGNSQV
SLC26A1       FSISLAEMFARSHGYSVRANQELLAVGCCNVLPAFLHCFATSAALAKSLVKTATGCRTQL

CG5404        ANLYLGIIVLLALSYLSPYFNYIPEATLAAILICSIFTLL-DFKLPMRLWRDSKRDFATW
CG6125        AGIYTGLIVLSALSILTPYFQYIPKASLSAVLIAAVIFMI-DLAPVKELWQTNKKDFFSW
CG6928        GGCYTSVLVLLALGLLAPYFQYIPKAALSAVIISAVIFMI-EFEVIKPLWRCSRRELLPG
SLC26A11      GGLVTGVLVLLSLDYLTSLFYYIPKSALAAVIIMAVAPLF-DTKIFRTLWRVKRLDLLPL
Neat          GGAVTGALVLMALAFLTQTFYFIPKCTLAAIIIAAMISLV-ELHKIKDMWKSKKKDLFPF
CG7005        SNIYSGGLVMIALLFLTPYFYFIPRPTLAAIIISAVVFMI-EVKVVKPMWRSKKSDLVPG
dPrestin      ASLVSASLVVVTLMWIGPFFSSLPRCVLAGVIIVALKPMFMQAKELKKFSKQGKLEMFTW
SLC26a6       AGAISSLFILLIIVKLGELFHDLPKAVLAAIIIVNLKGMLRQLSDMRSLWKANRADLLIW
SLC26A1       SSVVSATVVLLVLLALAPLFHDLQRSVLACVIVVSLRGALRKVWDLPRLWRMSPADALVW

CG5404        LLCFCVSVLFGVEVGLFVSIVVTALHLLFLWARPEIRVKIEQLDE--------------M
CG6125        VGSFIICLVAGVELGLLFGIVLSMVFILLRLGNPKFEVTLKQHES--------------T
CG6928        AITFVMSLAVGVEIGLLLGVSTDVAFLVYRAARPVLSVSKLQTTN--------------G
SLC26A11      CVTFLLC-FWEVQYGILAGALVSLLMLLHSAARPETKVSE--------------------
Neat          VVTVLTCMFWSLEYGILCGIGANMVYILYSSARPHVDIKLEKING--------------H
CG7005        VGTFVACLVLPLEWGILIGVGLNVIFILYHAARPKLSTELLTTQS--------------G
dPrestin      ISTFLCVVIIDIDIGLLIGICISLLALYIKGLKPYSCLLGYMPEAPGIYMDLNQHRNAMQ
SLC26a6       LVTFTATILLNLDLGLVVAVIFSLLLVVVRTQMPHYSVLGQVPDT-DIYRDVAEYSEAKE
SLC26A1       AGTAATCMLVSTEAGLLAGVILSLLSLAGRTQRPRTALLARIGDT-AFYEDATEFEGLVP


CG5404        -QYIRVTPSN-GIYFPAINYLRERVLKACEQ-A---------------------------
CG6125        -YYVHIVPQS-DVYYTGVDALRSELRGACRL-Y---------------------------
CG6928        INYILIRPKHSSLYFPAVEWVRSGISKALTI-----------------------------
SLC26A11      GPVLVLQPAS-GLSFPAMEALREEILSRALE-V---------------------------
Neat          -EVSVVDVKQ-KLDYASAEYLKEKVVRFLNNQN---------------------------
CG7005        VEYSMITPDR-CLIFPSVDYVRNLVNKQSIRQN---------------------------
dPrestin      VPEVRIFRYSGSLNFATSLFFRRALYEAVGLDKIPLTKVSSSNSNSPSK-----------
SLC26a6       VRGVKVFRSSATVYFANAEFYSDALKQRCGVDVDFLISQKKKLLKKQEQLKLKQLQKEEK
SLC26A1       EPGVRVFRFGGPLYYANKDFFLQSLYSLTGLDAGCMAARRKEGGSE-------------T
```

```
CG5404          --------------------------------------------------DFRITVVIDG
CG6125          --------------------------------------------------HNDFPVVLDC
CG6928          --------------------------------------------------HGTAPVVLDC
SLC26A11        --------------------------------------------------SPPRCLVLEC
Neat            --------------------------------------------------GETQLVVIKG
CG7005          -----------------------------------------------------VPVVIDA
dPrestin        GSKS-------SYS------------------PVSQNGGKAINGKLEETSGAFKVLVLDF
SLC26a6         LRKQAGPLLSACLAPQQVSSGDKMEDATANGQEDSKAPDGSTLKALGLPQPDFHSLILDL
SLC26A1         GVGEGGPA---------------------Q---GEDLGPVSTRAALVPAAAGFHTVVIDC

CG5404          QRISGMDYTAAQGISKLSSDLCRQADASSSTLLILFRFPEHLQRLIDNTDNLVFCESENK
CG6125          ARFMQFDATFSEMLISVAKEMASHD-----VLLILQNMSLKVQQMLPVMGNVRFCQEDSQ
CG6928          AHVHEFDFTAARGMGSLQKELAKAN-----APLFLMSADKTIGVILKESTNIDFPTIDCP
SLC26A11        THVCSIDYTVVLGLGELLQDFQKQG-----VALAFVGLQVPVLRVLLSADLKGFQYFS--
Neat            EEINSIDYTVAMNIVSMKGDLEALN-----CAMICWNWNIASAGVVCRLNNDLRPIFKFD
CG7005          SHVYGADFTTATVIDSLISDFNQRG-----QLLFFYNLKPSICSIFEHVSAAQFVVYYQE
dPrestin        SMLGHIDVAGCRTLTDLSKELKVRG-----ARLLLASPVDRVYDTLVHSMALSEGPF---
SLC26a6         GALSFVDTVCLKSLKNIFHDFREIE-----VEVYMAACHSPVVSQLEAGHFFDASI----
SLC26A1         APLLFLDAAGVSTLQDLRRDYGALG-----ISLLLACCSPPVRDILSRGGFLGEGPGDTA

CG5404          --VKEFLTQESLRNGYINLKEHIRASIDLGYKIDID--------------
CG6125          --LSSHLQ------------VEKEAPVVEAKL------------------
CG6928          DDLEFLLEQTA---D-YVLHLQISAPLVESRLVHGDSGEPTELSKLNGKSS
SLC26A11        -TLEEAEK------------HLRQEPGTQPYNIREDSILDQKVALLKA---
Neat            LSLEEVVA------------GHFDSPSNTASTVTIEA-------------
CG7005          QQLDELLK------------ERNYVQKRLETA------------------
dPrestin        ---EIFPTLHDC--VEYANACRTA--------------------------
SLC26a6         TKKHLFASVHDA--VTFALQHPRPVPDSPVSVTRL---------------
SLC26A1         EEEQLFLSVHDA--VQTARARHRELEATDAHL------------------
```
